# Echoes of the Month

**Published:** 1923-10

**Authors:** 


### ECHOES OF THE MONTH.

### DECREASE IN WOMEN MEDICAL STUDENTS.

Miss Brooks, the Warden of the London School of Medicine for Women, has been giving some reasons for the present decrease in the number of women taking up medicine as a career. For one thing, the post-war years had seen a set-back to women in all the professions, and the medical women students had had their special difficulties to contend with. Another reason was the critical financial position of the average middle-class household. In the last two years of the war and the two years following the Armistice there had been a great influx of women into the profession. Many of these women are passing out now fully qualified at the same time as the men who entered the schools on their return from the war. There is naturally a strong feeling among public bodies that the men who served should get the first chance of any positions there are to be offered, and this makes it difficult for women, especially for women outside London, to get posts. The cutting down of education grants has meant a decrease in the number of posts available for women as health inspectors in schools, but, on the other hand, there has been an increase in the demand for women to serve as inspectors of maternity and infant welfare work. There has not been an increase in the number of women going abroad, except for general practice.

### HEALTH METHODS THAT ARE TOO DIGNIFIED.

In the *Daily Telegraph* recently Dr. Addison has been comparing health publicity methods in America with those in England, greatly to the advantage of the former. "I do not advocate the use in this country of some of the blatant methods that have been tried in America, but far more might be done by use of the Press and the Poster in teaching the public some of the elementary rules of health. There is a great distinction between 'false publicity' that aims mainly at personal advertising and the true publicity that aims at arousing interest and educates the public to a fuller understanding of a given problem or set of problems. It must attract attention, or else it fails. But attention is not sufficient. Health publicity must arouse intelligent interest. But it is possible to be too dignified in our language, and personally I fear that much of the medical advice given in official reports in this country is written in an abstruse, technical style far above the heads of the semi-educated public."

### BABIES IN RUSSIA.

Russian doctors, says the Report of the International School of Nursing and Child Welfare for Russia, work sixteen hours a day, and are desperately few in number. One doctor to a population of 100,000 is no exaggeration. Sometimes he is in charge of as many as four hospitals forty miles apart. He generally has a few felchers (two years' medical students) to help him. The hospitals are few and overcrowded, and ill-equipped with the most simple and urgent necessities. The patients often lie two in a bed, and even on the floors, and they generally have to be nursed in their own clothing. In the Babies' Homes and Hospitals in the provinces there was often only a three-foot shelf arranged round the wall for the babies to lie on; no mattresses, no bed clothing. They were packed together like herrings. It was quite common to find dead children among the living. In some hospitals the beds had nothing but filthy rags on them, and owing to lack of soap these were never washed, but occasionally baked. Conditions in the surgical wards were appalling.

### THE HUMAN FLEA AS PLAGUE CARRIER.

An interesting suggestion has been made by Dr. William Lethem, Assistant Medical Officer of Health for the City and Port of Liverpool, in explanation of the reason why in the rare cases of bubonic plague attacking human beings in this country the disease tends to be confined to a single household, and to those residents who are in close touch with the patient. The doctor brings a charge of being *particeps criminis* in the matter against the human flea, which, he considers, has been ruled out as a direct cause on wholly insufficient evidence. He points out that in the outbreaks of plague in Great Britain since 1900 practically no plague rats were discovered, which absolves the rat flea from responsibility. On the other hand, the doctor concludes that the actual carrier of the pestilence is the human flea, or *pulex irritans*. In coming to this conclusion he relies especially on the Suffolk outbreak in 1910, when as many as seven cases occurred in one house in swift succession.

### DIET FOR CANCER PATIENTS.

Hope of alleviation from pain by means of a modification of diet was given to sufferers from cancer by Dr. S. Monckton Copeman in a lecture before the British Association. A diet from which foodstuffs of animal origin containing the fat-soluble vitamin were excluded was formulated from which was cut out everything containing animal fat, such as fat meat, butter, eggs, and cream; but bacon and ham were allowed to remain because the amount of fat-soluble factor they contain is exceptionally small. Latterly there had been added to this diet a little fat of vegetable origin. Cancer patients in infirmaries, etc., had, with their own consent, been put on this diet. The fat-solubles of vegetable origin are contained in lettuce, watercress, etc. Thanks to the co-operation of Archbishop Bourne, he had been able to study the statistics of the enclosed and the unenclosed communities of the Roman Catholic Church, and he found that, although the enclosed orders, who were practically vegetarians, had not complete immunity from cancer, at any rate they did not suffer so much in that respect as the unenclosed orders. Under the diet which had been prepared, which was satisfactory from the physiological point of view—full, varied, and appetising—it was found that patients increased in weight, and in some cases they had got freedom from pain, which was the only thing which would make life worth living.

### HEALTH PROPAGANDA AT A CARNIVAL.

In common with many other seaside resorts, Cleethorpes has recently been having a carnival. A fancy dress parade presented an excellent opportunity for the public health department to do a little propaganda work. There are ten schools in Cleethorpes, and the head teachers elected two scholars from each school, making ten boys and ten girls. The children were termed the health crusaders. On a frame-work running from end to end of a three-ton motor lorry was a hand-painted poster bearing the inscription, "The Cleethorpes School Children are the real Health Crusaders." Amongst the lettering large flies were painted. Around the motor body were fixed cartoons, etc., also posters bearing such inscriptions as "A smelling dust-bin is an indication of bad management." "The flies' circuit—from festering filth to food and fever." During the procession the girls, who were attired in fancy dress, were arranged five each side in the motor and armed with fly-swatters striking at the flies just above the level of their heads. The boys, also in fancy dress, walked five each side of the motor distributing hand-bills and selling the booklets "How we can assist to make an A1 nation," written by the school children.

### DETERIORATION IN CHILDREN'S STAMINA.

The annual report of the School Medical Officer for Monmouth states that, while, as a result of favourable economic conditions during the war, a general improvement in the physique and well-being of the children was observable, during the last three years a progressive deterioration has set in, and the advantage gained during the war period is gradually being lost. As most of the children now in school were born during the war years, this fact may have something to do with the change for the worse. But the evidence indicates the folly of the attempt to prevent the feeding of necessitous school children and the provision of adequate methods of relief. The clothing of the children is much worse now than in pre-war or war years.

### WHERE THERE ARE NO DECAYED TEETH.

In Porto Santo, a small island north west of Madeira, said Dr. Grabham at the British Association meeting at Liverpool, the healthy condition of the people's teeth is remarkable. Dentists and toothbrushes are unknown. He ascribed this to a great extent to the highly mineralised water springs. which analysis showed to be rich in many chemical salts,

A curious fact was that only soft food was eaten, and this in meagre quantities. Food was always taken cold, as also was the native form of coffee, and as the staple diet was porridge, made from maize, the teeth were never used for mastication. Meat and green vegetables were never eaten, but scurvy was unknown, and neither gastric disorders nor malignant disease could be discovered, though he had encountered cases of consumption. A remarkable feature was the appearance of a thin yellow line across the teeth in the upper jaw, which gradually spread and stained the teeth generally. His experience was that in Porto Santo the yellow stain was a sure indication of a sound set of teeth.

### CURE FOR SLEEPING SICKNESS.

Two German scientists, Professor Klein and Doctor Fischer, who have been testing the Bayer 205 treatment for sleeping sickness in Northern Rhodesia and the Congo, state that the results of their investigations had been most encouraging. The treatment had proved remarkably efficacious, and they claim that a hundred patients had been completely cured. Professor Klein said he believed that sleeping sickness, which afforded a very serious problem in North Rhodesia and the Congo, could be conquered by means of the Bayer treatment. Arrangements have been made for the continuance of experimental work at Bukama in the Congo. The scientists will now proceed to the Fernando Po Islands to carry out further experiments.

### HEALTHY FINLAND.

"The Finnish people are famous for their vigour and physique," writes the Medical Correspondent of *The Observer*. Here is a people typically rural in distribution and habits. "It is not fair," as the complaint has been made to me, "that you should compare the vital statistics of primitive rural peoples with those of typical modern cities." No question of fairness or unfairness arises; but such comparisons, if properly made, are eminently legitimate and desirable. The extraordinarily low mortality of infants in rural Finland, as in the rural parts of Ireland, may most properly be cited and studied whenever we are disposed to jubilation because our efforts and expenditure have brought some urban figures down to, say, only thrice those rural figures. I have been impressed by the extremely low consumption of alcohol among the Finns—not merely since they went "dry" a few years ago, but as long as any record goes back, and by the probable relation between this fact and their extraordinarily low infant mortality and high athletic prowess.

### THE DIRTIEST PLACE IN THE WORLD.

Although condemned thirty years ago as unfit for human habitation, the Salisbury Street area in Bermondsey still houses 1,000 persons, who will soon be turned out in order to demolish "the dirtiest place in the world." The houses are over 100 years old, and a number of them are back-to-back. The sickness, infantile and general mortality rates and tuberculosis rates are higher in this locality than anywhere else in London. The Borough Council have planned to erect artistic cottages in groups of two and four, with front and rear gardens. Each cottage will have three bedrooms, a parlour and a bathroom. Only 400 people can be accommodated where 1,000 now live, but accommodation for the remainder is promised by the conversion of an ex-workhouse into flats.

### OYSTERS CURE SCURVY.

The opening of the season has been celebrated at the French Academy of Science by the payment of high tribute to the medicinal and alimentary virtues of the oyster. This was a report by Madame Randoin, chief of the Institute of Food Hygiene, on the researches carried out by her under the instructions of the Director of Fishery Research. Her most important discovery is that oysters are a sure and rapid cure for scurvy, and that if only 15 grammes a day in summer, or less in winter, are eaten this quantity is sufficient to prevent scurvy. Her experiments were carried out on guinea pigs, which, having been dieted in such a way that they developed severe attacks of scurvy, were completely cured after being fed for a few days on oysters. Madame Randoin further reported that oysters eaten with lemon juice are an ideal form of food for sufferers from dyspepsia or debility, for oysters and lemon juice are both richer than any other food substance in vitamines.

